# Housing and Community Environments vs. Independent Mobility: Roles in Promoting Children’s Independent Travel and Unsupervised Outdoor Play

**DOI:** 10.3390/ijerph18042132

**Published:** 2021-02-22

**Authors:** Lingyi Qiu, Xuemei Zhu

**Affiliations:** Department of Architecture, College of Architecture, Texas A&M University, 3137 TAMU, College Station, TX 77843-3137, USA; xuemeizhu@tamu.edu

**Keywords:** children, independent mobility, physical environment, housing, neighborhood, independent travel, unsupervised outdoor play

## Abstract

Children’s independent mobility (CIM) has declined dramatically in recent decades despite its benefits in facilitating childhood development, promoting physical activity, and combating the obesity epidemic. This US-based study examines the impacts of housing and neighborhood environments on two modes of CIM—home-based independent travel to non-school destinations and unsupervised outdoor play—while considering personal and social factors. A bilingual parent/guardian survey was distributed to public elementary schools in Austin, Texas, asking about children’s travel and play, housing and neighborhood environments, and personal and social factors. A Google Street View audit was conducted to capture additional housing-related information. Logistic regressions were used to predict CIM. For second to fifth graders (*N* = 525), less than two-thirds of the parents would allow children’s independent travel to non-school destinations (62%) and unsupervised outdoor play (57.9%), with the majority limited to a short distance (five-minute walk) and a few destinations (e.g., friend’s/relative’s home). Stranger danger was a negative predictor and the presence of friend’s/relative’s home was a positive predictor for both modes of CIM. Quality of neighborhood environment was another positive correlate for independent travel to non-school destinations. Significant personal and social factors were also identified. Study findings demonstrated the impacts of physical environments on CIM and the potential of using relevant interventions to promote children’s health and development.

## 1. Introduction

Children’s independent mobility (CIM) refers to their freedom of moving around in the neighborhood without adult supervision or accompaniment [1]. It can be further categorized as independent travel or unsupervised outdoor play [2,3,4]. CIM is important to children’s physical, social, and cognitive development. Independent travel and unsupervised outdoor play can help children accumulate more daily physical activity [4,5,6], which is vital for their physical development such as motor skills, bone health, and weight control [5,7,8]. CIM can also facilitate children’s social and cognitive development. Children with greater independent access to neighborhood destinations were found to have more social interactions with their neighbors, better knowledge about their neighborhood and community [9,10], and better spatial and navigational skills [11]. Conversely, children with restricted independent travel and unsupervised outdoor play were reported to experience more negative feelings such as loneliness [12]. In addition, Prezza and Pacilli’s study [13] showed that greater autonomous mobility and more frequent play in public places could predict a stronger sense of community in adolescence. 

Despite these widely acknowledged benefits, CIM has declined across many developed countries over recent decades [14,15,16]. This decline also accounted for the overall decrease in children’s physical activity levels [5,17,18], which was closely linked with the obesity epidemic [19]. In a literature review article, Marzi and Reimers [18] recommended more intervention programs on CIM from the perspective of public health promotion, and highlighted the need for more high-quality studies exploring multilevel socioecological determinants of CIM to provide the necessary evidence.

Among different modes of CIM, independent school travel has been studied by researchers as a potential approach for children to accumulate physical activity through walking or cycling to/from school in daily routines [4,5,20,21,22]. Meanwhile, other modes of CIM, including independent travel to non-school destinations and unsupervised outdoor play, are largely understudied despite the fact that school days only account for about half of a year [4]. As suggested by Hillman [23], more studies are needed to better understand children’s travel and play activities during leisure time, because they may be significant sources of physical and social activities for children, especially on non-school days. 

Limited studies did examine the multilevel correlates for children’s independent non-school travel and unsupervised outdoor play. In terms of parental factors, significant results have been reported for parents’ socioeconomic status, age, gender, parenting style, education level, income, employment, occupation, and language proficiency [12,24,25]. Among personal factors, children’s age and gender were the most studied variables, and were significant in most relevant studies [12,26,27,28,29,30]. In addition, significant correlates of CIM have also been identified in the domain of social factors. For example, children’s independent travel to parks or shops was found to be less likely when social norms in parenting did not support such behaviors [24,31,32]. In contrast, stronger perceptions of social cohesion and neighborhood connection were reported to predict increases in children’s independent travel and unsupervised outdoor play, or the corresponding parental license for such behaviors (i.e., the distances that adults would permit for children’s independent travel and outdoor play) [24,32].

In addition to personal and social factors, housing and neighborhood environments are also likely crucial for CIM, as children are physically and mentally immature, and their mobility relies more on their immediate surroundings. Influences of physical environments on CIM are also likely domain/behavior specific, with different physical environmental factors being important to particular types of independent mobility. A limited number of studies have examined the role of physical environmental factors in independent non-school travel. Some studies explored the relationship between physical environments and independent travel to certain non-school neighborhood destinations such as friends’ homes, parks, shops, and recreation centers. Identified positive correlates included single-family housing and dense urban residential structures [33], while negative correlates included distance to destinations [31,34] and presence of alternative choices, such as the increasing access to local school grounds [31]. One study found that independent travel to local parks was less likely with increased distance to the closest parks and with better access to additional school grounds [31]. Another study reported that the increased distance to school also negatively affects the number of children’s independent non-school trips after school hours [32]. Higher floor–area ratios and a larger number of public transport hubs were also found to have negative influences on children’s independent non-school travel [33]. In addition, other environmental features such as traffic safety, sense of community, and stranger danger have also been reported to affect children’s home-based independent travel to non-school destinations [28]. Several other studies examined general independent travel without specifying the destinations. They also reported a few significant environmental correlates for this mode of CIM. A meta-analytic review reported that four physical environmental factors—dead-end streets, percentage of residential land uses, percentage of commercial land uses, and residential location type (urban–suburban)—had positive associations with independent travel, while vehicular street width, road density, intersection density, major road proportion, land use mix, availability of recreational facilities, residential density, and distance to destinations are negative correlates [34]. In addition, increased urbanization was found to be associated with decreased independent travel among children [28]. 

Furthermore, a very limited number of studies specifically focused on children’s “unsupervised” outdoor play in their neighborhood. Therefore, we broadened the scope of our review on this topic by including studies on the impacts of neighborhood and housing environments on any outdoor play activities (unsupervised or not) in the neighborhood. For this more general category of outdoor play, positive correlates on neighborhood level included presence of sidewalks, presence of traffic-safety-related features (e.g., pedestrian crossings, traffic lights, speed bumps, parallel parking spaces, grouped parking lots, home zones, and roundabouts) [35], presence of green spaces [36,37], having a yard near home to play in [38], and presence of cul-de-sacs in the neighborhood [36]. In contrast, the presence of intersections, street lighting, the greater number of formal outdoor play facilities [35], and GIS-derived main street density in a 100-m buffer around a child’s home in more urbanized areas [29], were found to be negatively related to children’s outdoor play. In addition to these neighborhood features, housing characteristics also showed significant impacts on children’s outdoor play. For example, one study examined outdoor play among children in different gender and age groups and reported the better maintenance of homes in the neighborhood to be negatively related to outdoor play among boys aged 10–12 years [35]. 

Overall, there are limited studies about children’s independent non-school travel or unsupervised outdoor play that are domain/behavior specific. In addition, findings from different studies are not always consistent, which may be partially due to the diverse study contexts and the varying definitions and measures of CIM used in these studies. Furthermore, most recent CIM studies are based on the settings in Europe, Canada, Australia, and New Zealand [18,39,40,41]. Only a few studies on CIM were conducted in the US and examined the relevant personal, social, and physical environmental factors. Thus, it is essential to further understand the impacts of personal, social, and physical environmental factors on CIM in the US.

Aiming to bridge the aforementioned knowledge gaps, this US-based study examines the impacts of housing and neighborhood environments on two modes of CIM—children’s home-based independent travel to non-school destinations and unsupervised outdoor play in home neighborhood—in the City of Austin, Texas, while taking individual and social factors into consideration. Physical environmental factors such as stranger danger and the presence of friend’s or relative’s home were found to be significant predictors of CIM, while other significant personal and social correlates were also identified. The findings of this study showed the impacts of physical environments on CIM, identified the potential of relevant interventions for future studies, and suggested practical implications to help promote CIM and children’s health.

## 2. Methods 

### 2.1. Conceptual Framework

**Figure 1:**
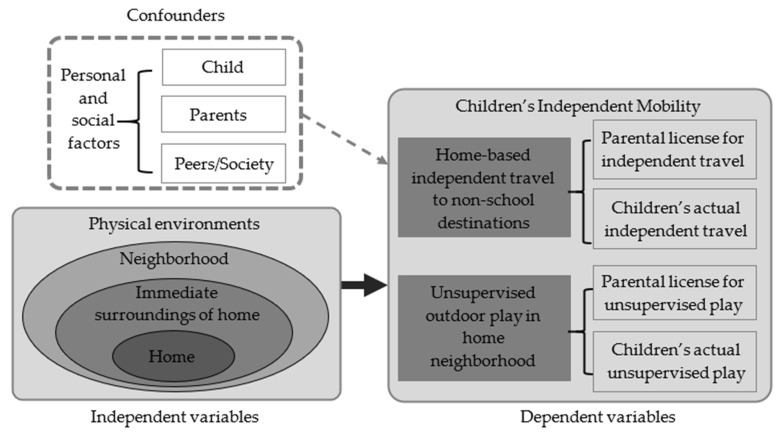
Conceptual framework about multilevel factors affecting two modes of children’s independent mobility.

The social–ecological model [42] is frequently used to guide research on human behaviors and relevant environmental interventions from multiple levels, including intrapersonal factors, interpersonal processes and primary groups, institutional factors, community factors, and public policies. This study proposed a conceptual framework about CIM (Figure 1) based on the Social–Ecological Model for Child’s Development [43], which emphasizes the significance of children’s immediate environments to their development. This is grounded in the consideration that a child’s biological and psychological makeup is based on individual and genetic developmental history, but also continues to be affected and modified by the child’s immediate physical and social environment (microsystem), as well as interactions among the systems within the environment (mesosystems) [43,44]. The proposed conceptual framework considers multilevel factors affecting two modes of CIM, including personal and social factors, as well as physical environmental factors of an individuals’ home, the immediate surroundings of the home, and the surrounding neighborhood.

### 2.2. Study Design

This cross-sectional study focuses on students attending public elementary schools in the Austin Independent School District (AISD) or living within the City boundary of Austin, Texas, and their parents/guardians. All the study materials and protocols were reviewed and approved by the Institutional Review Board at the researchers’ institution (IRB2018-0270D).

### 2.3. Study Setting and Population

**Figure 2:**
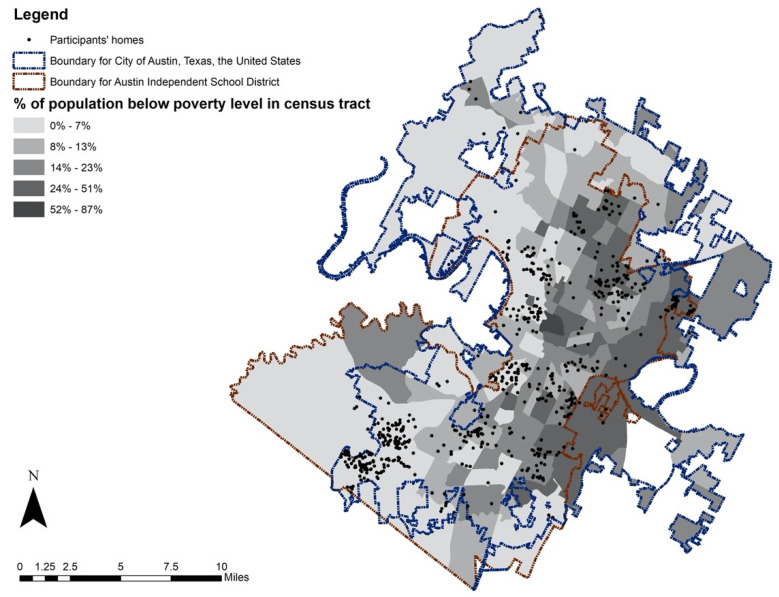
Study area and home locations of study participants.

The study setting consists of the AISD and a small area that is not in AISD but within the boundary of the City of Austin (Figure 2). This area has a diverse population with varying sociodemographic characteristics and diverse neighborhood environments. In the 2018–2019 academic year, AISD had an enrollment of 79,787 students, and more than half (55.5%) of them were Hispanic; the rate of economically disadvantaged students (i.e., a student who is eligible for free or reduced-price meals under the National School Lunch and Child Nutrition Program) was 53.5% [45]. Among 42,599 students from the total of 87 public elementary schools in AISD, 56.1% of them were Hispanic (*n* = 23,877), and 57% of the total students were eligible for free or reduced-price lunch (*n* = 24,297).

The study population is second to fifth graders from the study area and their parents or guardians. Children in the second to fifth grade were chosen because of their specific characteristics at this unique developmental stage. Based on Piaget’s theory [46], children of seven and older start trending toward autonomy while also becoming more socialized than in early childhood [28]. Students in the second to fifth grade are right at this stage and have already accumulated a certain ability to operate actions in reality, developed some autonomy, and increased socialization to some degree—which are basic skills for the fulfillment of independent travel or unsupervised play. On the other hand, second to fifth graders are just starting to explore outdoor environments independently and thus are highly dependent on environmental support. Hence, this study included second to fifth graders from elementary schools in the study area as the study sample to investigate CIM and the corresponding multilevel correlates.

### 2.4. Data Collection

A bilingual parent/guardian survey was developed and distributed to collect information about children’s travel and play activities, housing and neighborhood environmental factors, as well as personal and social factors. The survey was created based on two validated survey instruments—the Safe Routes to School Survey [47] and the Neighborhood Environment Walkability Scale (NEWS)-Youth Survey [48], as well as findings from the researchers’ literature review [39]. During the survey instrument development process, cognitive interviews were conducted to facilitate the testing and finalization of the instrument, with a convenient sample of ten participants recruited from the researchers’ institution. Considering the high proportion of the Hispanic population in the study area, the research team also developed a Spanish version of the survey through a two-way translation process.

The survey data were collected between November 2018 and July 2019. In November 2018, with support from the City of Austin’s Safe Routes to School Program, hard copies of the surveys were delivered to 24 public elementary schools in the AISD, which represent the diverse sociodemographic characteristics and neighborhood environmental features in the study area. Participants were invited to either complete the paper survey and return it to their child’s schoolteacher or take the survey online using the link provided in the cover letter sent along with the paper survey. Later on, two rounds of survey reminders were posted in local online neighborhood forums—NextDoor—in late May and June of 2019 to solicit more survey responses. The data collection process was closed at the end of July 2019.

After the completion of the survey, additional data about participants’ homes were collected according to the home address reported in the survey. The information about housing type for each participant’s home was extracted from the public appraisal data requested from the Central Appraisal District of Travis County and Williamson County. The Walk Score, Bike Score, and Transit Score of each home location were obtained from the Walk Score^TM^ website (https://www.walkscore.com/ accessed on 18 February 2021) (Walk Score, Seattle, WA, USA). In addition, a Google Street View (GSV) (Google LLC, Menlo Park, CA, USA) audit was conducted to collect information for several additional housing-related physical environmental variables, including the presence of a front yard, backyard, driveway, or frontage street for the participants’ homes, and the presence of apartment common areas around the homes. 

### 2.5. Measures

This study’s two outcome variables—children’s home-based independent travel to non-school destinations and unsupervised outdoor play in neighborhood—were measured through parents’ or guardians’ report of (1) mobility “license,” which refers to their permission for their child to independently travel and play without adult supervision, and (2) their child’s actual fulfillment of independent mobility. Two specific questions were asked in the survey to capture mobility license, including “how far away from home is your child allowed to go without adult accompaniment?” and “how far away from home is your child allowed to play in outdoor areas without adult accompaniment?” Six options in terms of children’s mobility ranges were provided, including “never allowed,” “less than 5 min walk,” “6–10 min walk,” “11–15 min walk,” “16–20 min walk,” and “more than 20 min walk.” Furthermore, in order to measure the actual fulfillment of independent mobility, parents or guardians were asked to select neighborhood destinations that their children actually independently travel to, and to report the daily average time that children spent on unsupervised outdoor play in total and at each neighborhood location (e.g., school, park, playground) or place directly near their homes (e.g., own yard, own driveway, frontage street). 

Items from the validated instrument NEWS-Youth Survey [48], and questions developed based on the researchers’ literature review were used in the survey to measure participants’ housing and neighborhood physical environments as well as social factors. Participants responded to those questions by choosing from four-point Likert-scale answers, ranging from “1 = strongly disagree” to “4 = strongly agree,” to assess the quality of neighborhood environments such as access to services, neighborhood surroundings, neighborhood safety, and crime safety, and social factors such as neighborhood social connection, neighborhood social support, and neighborhood social norms.

The accuracy of housing type information from the survey was further checked and validated by referring to the public appraisal data from the County’s Appraisal Central District, because some respondents seemed to be unclear about the definitions for certain housing types, such as a one-family house attached to one or more houses (e.g., townhouse) and a building with two to four apartments or units (e.g., duplex, fourplex). When there was an inconsistency between the survey response and the record in the appraisal data, the housing type information from the appraisal data was used in the final analyses. GSV audits were also conducted to further ensure the accuracy of those homes’ housing types and collect housing type information for a few homes missing the relevant information in the appraisal data. In addition, GSV audits were conducted to further validate a few other housing-related variables, including having one’s own front yard, backyard, driveway, or frontage street, or having apartment common areas near homes. For 79 (15%) participants who did not provide a valid home address, all housing-related variables were based on their survey responses.

Furthermore, Walk Scores, Bike Scores, and Transit Scores of participants’ home locations were obtained from the Walk Score^TM^ website (https://www.walkscore.com/ accessed on 18 February 2021). Walk Score^TM^ is a website that provides scores on a scale from 0–100 to estimate walkability for a given location (Walk Score), as well as whether a location is good for biking (Bike Score) and well served by public transit (Transit Score). The Walk Score has been identified as a reliable and valid tool to measure neighborhood walkability [49,50], especially access to walkable amenities [51]. The Transit Score has also been demonstrated to be a valid measure of transit availability [50,52]. Validity of Bike Scores still needs to be further tested, but some recent studies did showcase the correlation between bikeability and cycling behavior [53], cyclist safety [54], and other cycling-related urban and human issues [55].

A series of sociodemographic variables were reported by parents or guardians in the survey. The information collected about children included grade level, gender, ethnicity, health conditions, and the eligibility for free or reduced-price lunch. The collected factors related to parents/guardians or the household included the parents’ highest education and occupation, home language, years lived in the current residence, home ownership, household car ownership, dog ownership, and parents’ negative attitudes toward CIM.

### 2.6. Statistical Analyses

Descriptive statistics were reviewed for all study variables, and some variables were recoded because of the highly skewed distribution. The two outcome variables about parental license for children’s home-based independent travel and unsupervised outdoor play were recoded as binary variables, 0 = never allowed and 1 = allowed, as responses to these two questions were highly skewed with a higher portion of “never allowed.” Additionally, due to the highly skewed distribution of the original values, the Walk Score for each home (scale from 0–100) was recoded as 1 = almost all errands car dependent (0–24), 2 = most errands car dependent (25–49), and 3 = somewhat walkable to very walkable (50–100). A similar scheme was applied for recoding the Bike Score and Transit Score. For Likert-scale variables from NEWS items and questions developed based on the researchers’ literature review, the percentage of missing data ranges from 1.5% to 4.8%, and means were used for imputing missing data. 

Two binary logistic regression models were used to predict two outcome variables— independent non-school travel and unsupervised outdoor play, respectively. The predictors included in the regression models were selected based on the findings from the literature review and relevant theoretical basis, and they are slightly different between the two models. For example, the model for unsupervised outdoor play included additional housing-related variables, such as having their own driveway or frontage street, which were not included in the model for predicting independent non-school travel, as these variables are not directly relevant to travel behaviors. 

Factor analyses and bivariate tests were used to guide variable reduction. For variables about neighborhood environments, factor analysis was applied, and six factor variables were generated, including neighborhood support and positive impacts from peers, stranger danger, quality of neighborhood surrounding environments, crime danger, sidewalk availability and buffer from street, and access to services. Children’s personal factors, including grade level, gender, ethnicity, health conditions, and social factors, were included in the final multivariate binary logistic regression due to their theoretical importance. For other independent or confounding variables, the bivariate relationship with each outcome variable was tested by binary logistic regression analysis. Only those with significant bivariate relationships with the outcome variable (*p* < 0.05) were retained for the final multivariate binary logistic regression to examine the association between housing and neighborhood environmental factors and CIM while considering personal and social factors. Analyses were performed using SPSS Statistics 27 software (IBM Corporation, Armonk, NY, USA).

## 3. Results

### 3.1. Descriptive Statistics

A total of 525 valid survey responses were received from parents or guardians of second to fifth graders in the study area and are included in this analysis for this paper. Characteristics of children included in analyses are provided in Table 1. A little less than half of them (48.3%) were girls, and the average grade of the study sample was 3.3. Overall, 40.2% of them were Hispanic, and 34.9% were eligible for free or reduced-price lunch (Table 1). Compared to the study population, this study sample has a relatively smaller percentage of students who were Hispanic and who were eligible for free or reduced-price lunch.

**Figure 3:**
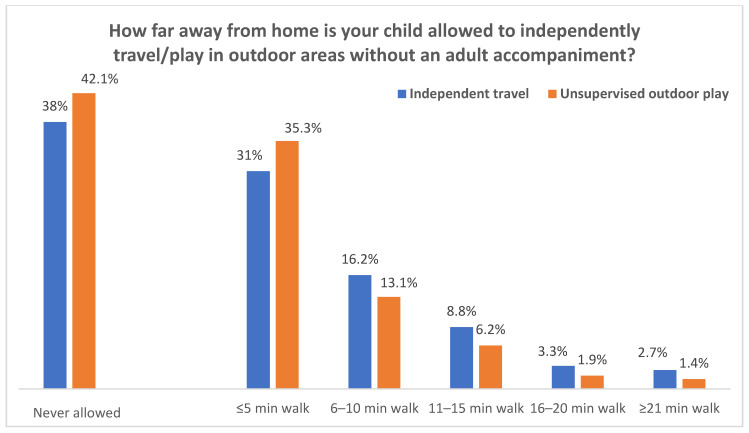
Parental license for independent travel and unsupervised outdoor play (second to fifth grade).

**Figure 4:**
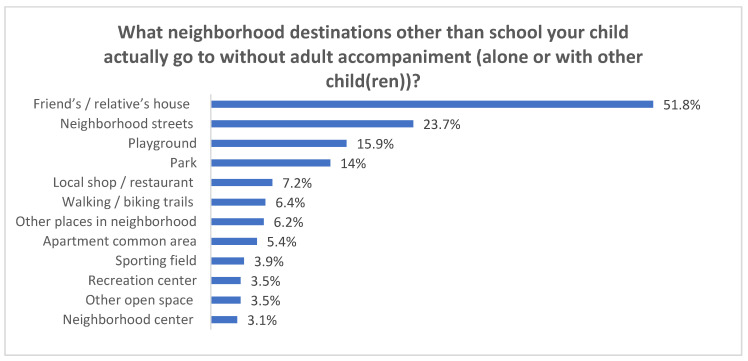
Non-school neighborhood destinations that children actually independently travel to (*n* = 515).

The proportions of parental license to children’s home-based independent non-school travel and unsupervised outdoor play by children’s grades are shown in Figure 3 and Figure 4. Overall, for second to fifth graders, 62% of the parents would allow children’s home-based independent travel to non-school destinations, and the percentage of parents who would permit home-based unsupervised outdoor play in the neighborhood was 57.9% (Figure 3). However, most of the allowed travel or play was limited to a very short distance (Figure 3) and a few destinations (Figure 4). For example, 31% of parents would only allow their child to independently travel to non-school destinations within a five-minute walk distance from home, and this accounted for 50% of all allowed home-based independent travel to non-school destinations. Similarly, 35.3% of parents would only allow their children’s unsupervised outdoor play within a five-minute walk from home, and this accounted for 61% of all allowed unsupervised outdoor play.

The most popular neighborhood destination that children actually independently traveled to was a friend’s or a relative’s home within the neighborhood (Figure 4). More than half (51.8%) of participants’ parents or guardians reported a friend’s or a relative’s home as the destination for their children’s actual independent travel. Other popular neighborhood destinations included neighborhood streets, playgrounds, parks, etc.

**Figure 5:**
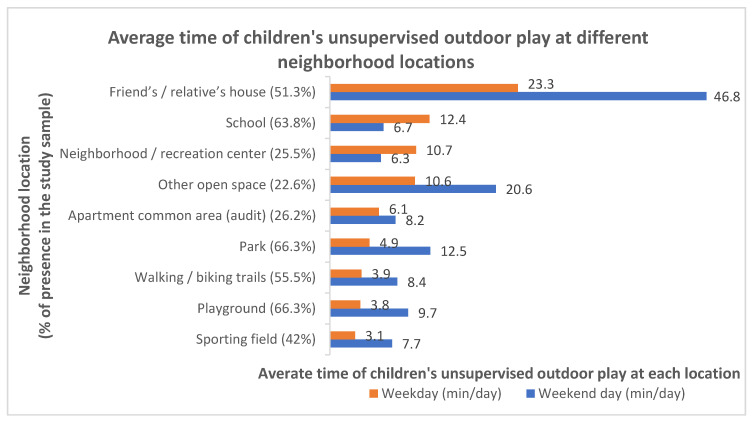
Percentages of participants with specific neighborhood amenities and among them the amount of time their child spent for unsupervised play at each location.

In order to better understand the spatial and temporal patterns of unsupervised play, the survey also asked about the availability of specific neighborhood amenities and places around homes, and how much time the child typically played there on a weekday or weekend day without adult supervision. Figure 5 illustrates the percentages of participants having these neighborhood amenities, and among them, how much time their child spent for unsupervised play at each destination. Among participants who had these specific amenities in their neighborhood, a friend’s or a relative’s home was where children spent most of their time playing both on a weekday (an average of 23.3 min/day) and a weekend day (an average of 46.8 min/day). For other locations, the popularity varied between weekdays and weekend days, with schools, neighborhood/recreation centers, and other open spaces being the most popular places following a friend’s/relative’s house on weekdays, and with other open spaces, parks, and playgrounds being the most popular locations following a friend’s/relative’s house on weekend days. Time spent on unsupervised play was in general longer during weekend days.

**Figure 6:**
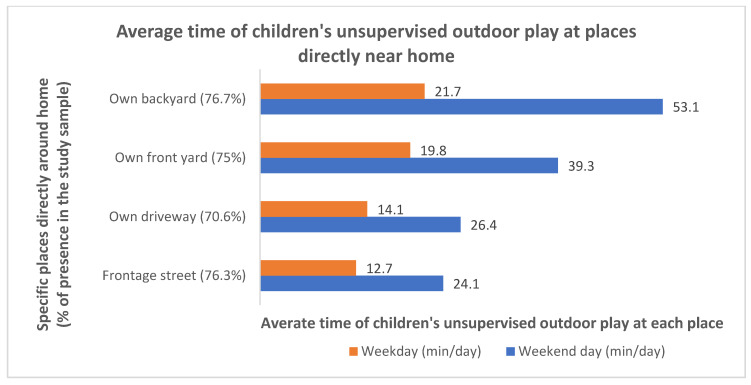
Percentages of participants with specific places directly near home and among them the average time their child spent at each place for unsupervised outdoor play.

The survey also asked about the presence and use of specific places directly near home for unsupervised outdoor play. The percentages of participants with their own backyard, front yard, driveway, and frontage streets were 76.7%, 75%, 70.6%, and 76.3%, respectively (Figure 6). Among families having these places, children spent the most time playing in their own yards, especially backyards, without adult supervision. For those with backyards, parents reported an average of 21.7 min that their child spent playing there without supervision on a weekday, and an average of 53.1 min on a weekend day. In contrast, children spent slightly less time in their front yards than in backyards, with an average of 19.8 min on a weekday and 40.2 min on a weekend day. 

### 3.2. Results of Binary Logistic Regression Analyses

Table 2 shows the results of the adjusted binary logistic regression models predicting two CIM outcomes. Model 1 predicts the likelihood for parental license for children to independently travel from home to non-school destinations. Results showed a child’s higher grade level (odds ratio (OR) = 1.423, 95% confidence interval (CI) = 1.091, 1.855) and the number of years lived in current residence (OR = 1.206, 95% CI = 1.026, 1.418) were significantly associated with the increased likelihood of parental license for this behavior, while children’s number of health conditions (OR = 0.613, 95% CI = 0.380, 0.991) or parents’ negative attitude toward independent travel (OR = 0.533, 95% CI = 0.425, 0.667) were significantly associated with the reduced odds to be allowed to do so. None of the social factors were significant at *p* < 0.05 level in predicting parental license for children’s home-based independent travel to non-school destinations. For physical environments, the presence of a friend’s or a relative’s home in the neighborhood (OR = 2.651, 95% CI = 1.471, 4.779) and quality of surrounding neighborhood environments (OR = 1.389, 95% CI = 1.015, 1.902) were found to have a significant positive impact on parental license to their children’s independent travel, while stranger danger (OR = 0.555, 95% CI = 0.408, 0.757) played a significant negative role. Some other factors were found to be marginally significant in predicting the odds of parental license to independent non-school travel (0.05 ≤ *p* < 0.1), including the factor variable for neighborhood support and positive peer influences (marginally positive) and eligibility for free or reduced-price lunch and the presence of walking/biking trails in the neighborhood (marginally negative).

Model 2 predicts the parental license for unsupervised outdoor play in home neighborhood using multilevel factors. The number of child’s health conditions (OR = 0.551, 95% CI = 0.337, 0.901) or parents’ negative attitude toward unsupervised outdoor play (OR = 0.395, 95% CI = 0.288, 0.540) were associated with reduced likelihood to allow their children to play outdoors independently. The social factor of strong neighborhood support and positive peer influences (OR = 2.285, 95% CI = 1.560, 3.348) was a positive correlate. Furthermore, among neighborhood physical environmental factors, the presence of a friend’s or a relative’s home (OR = 2.210, 95% CI = 1.240, 3.937) increased the likelihood for parents to allow children’s unsupervised outdoor play, while stranger danger (OR = 0.535, 95% CI = 0.390, 0.732) was a significant negative predictor. Higher grade level and better quality of surrounding neighborhood environments were marginally associated with increased likelihood for parents to allow children’s unsupervised outdoor play (0.05 ≤ *p* < 0.1). 

## 4. Discussion and Implications

This study used data from parents’/guardians’ survey responses, GSV audits, and other public data sources (e.g., county’s Central Appraisal District, Walk Score website) to investigate the association between housing and neighborhood environments and two modes of CIM, including home-based independent travel to non-school destinations and unsupervised outdoor play in home neighborhood while taking personal and social factors into consideration. For our study sample—second to fifth graders attending public elementary schools in the City of Austin, Texas, USA—less than two-thirds were allowed to have a certain extent of home-based independent travel to non-school destinations and unsupervised outdoor play, and these were mostly limited to a short distance of five-minute walk (around 0.25 mile) from home. As the distance grows, the percentage of allowed CIM decreased dramatically, likely due to the increased concerns about children’s safety as they play or travel further away from home. Though not exactly the same, the findings of parental license to CIM range are similar to an earlier study that reported 62% of parents of children 8–12 years old would restrict their independent travel to places within 500 m (approximately 0.31 mile) from home and 74% would restrict unsupervised outdoor play to within the same range [25]. Our study also found out that the most popular neighborhood destination that children actually independently travel to was a friend’s or a relative’s home in the neighborhood. It was far more prevalent than the second popular place, neighborhood streets (51.8% vs. 23.7%). The findings are consistent with earlier studies that also identified a friend or a relative’s home in the neighborhood as the most frequently visited destination of a child’s independent non-school travel [56,57]. This present study also reported that a friend’s or a relative’s home was the neighborhood location where children spent the most time playing without adult supervision on both weekdays and weekend days. Meanwhile, participants’ own yards (backyard and front yard) were the places directly near home where children spent the most time playing without adult supervision. A similar finding that children’s own home yard was the most frequently reported location for active free play was identified by a qualitative study investigating children’s free time play by interviewing parents [58]. 

The findings of this study indicate that there might not be sufficient safe and attractive neighborhood destinations/places where children can independently travel to or play without adult supervision, and the existing neighborhood destinations/places may lack child-friendly features and safety that can make parents more comfortable about allowing their children’s independent travel or unsupervised play. According to Chatterjee’s studies [59,60], a child-friendly place should have the following qualities or affordances: “(1) providing opportunities for children to develop an attitude of care for places that children love and respect; (2) promoting meaningful exchange between child and place through affordance actualization in places; (3) offering opportunities for environmental learning and developing environmental competence through direct experience in places; (4) allowing children to create and control territories and protect these territories from harm; (5) providing privacy experiences and nurturing childhood secrets; and (6) allowing children to express themselves freely in place.” More studies about child-friendly neighborhood destinations/places are needed to fully address parental barriers, inform relevant design interventions, and promote CIM.

Stranger danger was a negative predictor for both independent travel and unsupervised outdoor play. Several concepts and guidelines might be applied to address this issue through design. One is to create Defensible Space, which operates by dividing large neighborhood public spaces and assigning them to individual and small groups to use and enhance the users’ sense of control of the space and thus help prevent and reduce stranger danger [61,62,63]. Another applicable concept is Crime Prevention through Environmental Design (CPTED), which emphasizes creating safer neighborhoods through built environments and design strategies of territoriality, surveillance, access control, and maintenance [64]. For example, more surveillance could be provided along neighborhood streets and other open spaces by designing housing with more windows facing those areas.

The factor variable for quality of neighborhood surrounding environments was a significant predictor for children’s home-based independent travel, and had a marginally significant impact on children’s unsupervised outdoor play in their home neighborhood. Several design strategies can be considered to help create neighborhood environments with satisfying quality. One example is providing substantial green spaces for plants and small animals, thereby encouraging children to engage in outdoor activities while facilitating their learning from nature. This strategy was also proposed in the framework created by the United Nations International Children’s Emergency Fund (UNICEF) for defining and guiding the development of a “Child-Friendly City” [65] and other studies on creating child-friendly environments [66,67,68]. Providing more child-friendly amenities and infrastructure in the neighborhood can help guarantee every child’s right to use the neighborhood environments. Another example is to use buffers between sidewalks and roadways as well as protected bike lanes to help ensure children’s traffic safety and thus encourage their independent travel and play along the streets. Furthermore, spaces should be designed and created to meet children’s diverse needs for activities, privacy, and socializing. Samples of relevant considerations include comfortable dimensions and scales, purpose-built play areas to accommodate different age groups’ diverse play activities, and yards and balconies for multifamily housing to maximize parental license to children’s unsupervised outdoor play. Last but not least, in addition to solely focusing on the perspective of built environments, actions should be taken from multiple aspects to create child-friendly communities. A recent study proposed a Dynamic Human Ecology Framework for Healthy Places for Children and suggested that collaboration, inclusion, and engagement were key to creating child-friendly communities, and collaborative efforts should be provided from multiple levels, including local government, health providers and schools, social inclusion, and family and children [69]. 

One of the main limitations of this study is its cross-sectional design. Although our results showed correlations between personal, social, and physical environmental factors and CIM, this study cannot assess causality. Another limitation is that we used parents’ reported license for children’s independent mobility as the outcome variables in the multivariable analyses, which may not fully and precisely represent children’s actual independent mobility compared to objectively measured travel trips and play time. Some of the physical environment variables, including the presence of neighborhood amenities such as schools, playgrounds, and parks, were from self-reported survey responses, which would probably result in certain inaccuracy of the data. The research team is currently in the progress of collecting additional objective data for housing and neighborhood environments using Geographic Information System (GIS) and GSV audits. It is expected that the results based on objective environmental data will help reduce the potential self-report bias, help further understand the impacts of physical environments on CIM, and inform design and planning practice. 

## 5. Conclusions

In summary, this study explored the current status for two types of CIM—home-based independent travel to non-school destinations and unsupervised outdoor play in home neighborhood—among second to fifth graders attending public elementary schools in Austin, Texas, USA. We also examined the correlation between participants’ housing and neighborhood physical environments and two modes of CIM, while accounting for personal and social factors. The results showed that children were less likely to be allowed to travel to non-school neighborhood destinations independently if they are younger, have health conditions, have lived in their current residence for fewer years, or have parents/guardians with a negative attitude toward independent travel behavior. Meanwhile, the likelihood of independent travel is higher when there is a friend’s or a relative’s home in the same neighborhood, better quality in surrounding environments, and less stranger danger in the neighborhood. Furthermore, children were more likely to play outdoors unsupervised if they have fewer health conditions, a friend’s or a relative’s home in their neighborhood, and parents with more positive attitudes toward unsupervised outdoor play, as well as if there is less strange danger, better quality of surrounding environments, and more neighborhood support and positive peer influences. The findings suggested the potential of using targeted environmental interventions to encourage children’s independent travel and outdoor play and thereby promote children’s development, improve children’s physical activity, and combat the obesity epidemic. Meanwhile, identified essential housing and neighborhood environmental features could be developed into operational design strategies and contribute to current conceptual frameworks and guidelines for creating child-friendly environments. The study findings can also be informative to policy-makers, planners, or architects in guiding future housing and neighborhood programs to create more child-friendly environments.

## Figures and Tables

**Table 1:** Sociodemographic characteristics of the study sample

Sociodemographic Characteristics		N (%) or Mean
Child grade		3.3 (range: 2–5)
Child gender (female)		251 (48.3%)
Race/ethnicity	African American	12 (2.4%)
	Hispanic	201 (40.2%)
	White, non-Hispanic	235 (47%)
	Other	52 (10.2%)
Eligible for free/reduced-price lunch		180 (34.9%)
Total study sample		525

**Table 2:** Binary logistic regressions predicting parental license for independent travel to non-school destinations and unsupervised outdoor play using personal, social, and physical environment factors

Predictors	Coding Scheme or Range of Factors	Odds Ratio (95% Confidence Interval)	
		Model 1: Predicting Parental License For Independent Travel to Non-School Destinations (*N* = 439)	Model 2: Predicting Parental License for Unsupervised Outdoor Play (*N* = 446)
**Child personal factors**			
Child’s gender (Male %)	0 = female, 1 = male	0.773 (0.447, 1.335)	1.169 (0.678, 2.013)
**Child’s grade level**	0 = kindergarten, 1 = first grade…, 5 = fifth grade	1.423 (1.091, 1.855) **	1.290 (0.995, 1.673) ^†^
Child’s ethnicity (Hispanic %)	0 = non-Hispanic, 1 = Hispanic	1.253 (0.573, 2.741)	0.935 (0.426, 2.052)
Eligibility for free or reduced-price lunch	0 = no, 1 = yes	0.408 (0.165, 1.010) ^†^	1.896 (0.717, 5.012)
**Child’s health conditions**	The total number of health conditions a child has	0.613 (0.380, 0.991) *	0.551 (0.337, 0.901) *
**Parental and household factors**			
Parent occupation	0 = unemployed, 1 = employed	0.864 (0.439, 1.699)	1.427 (0.742, 2.744)
English as home language	0 = no, 1 = yes	1.886 (0.801, 4.438)	1.205 (0.508, 2.855)
**Year(s) lived in current ** **residence**	1 = <2 years; 2 = 2–<4 years; 3 = 4–<6 years; 4 = 6–<8 years; 5 = 8–<10 years; 6 = 10 years or longer	1.206 (1.026, 1.418) *	1.072 (0.914, 1.257)
Home ownership	0 = rent, 1 = own	1.148 (0.440, 2.996)	1.310 (0.478, 3.593)
Household’s car ownership	Number of motor vehicles in the household	1.294 (0.818, 2.047)	0.870 (0.562, 1.349)
Dog ownership	0 = no, 1 = yes	0.664 (0.371, 1.186)	0.656 (0.359, 1.197)
**Parent’s negative attitude to independent travel**	1 = strongly disagree…, 4 = strongly agree	0.533 (0.425, 0.667) ***	N/A
**Parent’s negative attitude to unsupervised outdoor play**	1 = strongly disagree…, 4 = strongly agree	N/A	0.395 (0.288, 0.540) ***
**Social factors**			
Social connection—“I feel connected to people in my neighborhood.”	1 = strongly disagree…, 4 = strongly agree	0.986 (0.713, 1.364)	0.873 (0.635, 1.200)
**Neighborhood support and positive peer influences**	Factor (Range: −3.020, 2.276)	1.365 (0.943, 1.976) ^†^	2.285 (1.560, 3.348) ***
**Physical environmental factors**			
Housing type x have own yard (reference: non-single-family without own yard)	0 = no, 1 = yes		
Non-single-family but have at least one own yard		1.376 (0.405, 4.684)	0.589 (0.138, 2.508)
Single-family housing		0.824 (0.179, 3.799)	0.271 (0.032, 2.326)
Presence of…around home	0 = no, 1 = yes		
Own driveway		N/A	2.627 (0.656, 10.526)
Frontage street		N/A	2.476 (0.679, 9.027)
Presence of…in neighborhood	0 = no, 1 = yes		
School		1.449 (0.766, 2.742)	N/A
Playground		0.764 (0.379, 1.541)	1.124 (0.585, 2.160)
Walking/biking trails		0.515 (0.263, 1.009) ^†^	0.747 (0.397, 1.406)
**Friend’s/relative’s house**		2.651 (1.471, 4.779) **	2.210 (1.240, 3.937) **
Apartment common areas		1.641 (0.498, 5.405)	1.144 (0.323, 4.051)
Other open space		1.053 (0.534, 2.078)	N/A
**Quality of surrounding ** **neighborhood environments**	Factor (Range: −3.489, 2.343)	1.389 (1.015, 1.902) *	1.367 (1.000, 1.868) ^†^
**Stranger danger**	Factor (Range: −2.968, 2.477)	0.555 (0.408, 0.757) ***	0.535 (0.390, 0.732) ***
Sidewalk availability and condition	Factor (Range: –3.042, 2.239)	N/A	0.985 (0.725, 1.339)
Crime danger	Factor (Range: −3.044, 3.747)	0.882 (0.663, 1.173)	N/A
Walk Score (reference: almost all errands car dependent)	1 = all most all errands car dependent; 2 = most errands car dependent; 3 = walkable		
Most errands car dependent		1.466 (0.656, 3.275)	0.999 (0.452, 2.208)
Walkable		0.954 (0.384, 2.367)	1.143 (0.458, 2.852)
Transit Score (reference: minimal transit)	1 = minimal transit; 2 = some transit; 3 = good transit		
Some transit		0.615 (0.272, 1.390)	0.526 (0.234, 1.180)
Good transit		0.349 (0.094, 1.260)	0.506 (0.133, 1.933)
		Cox & Snell R Square: 0.397, Nagelkerke R Square: 0.543	Cox & Snell R Square: 0.425, Nagelkerke R Square: 0.573

^†^*p* < 0.1; * *p* < 0.05; ** *p* < 0.01; *** *p* < 0.001.

## Data Availability

The data are not publicly available due to the need to protect the privacy of research participants.
